# Influenza Vaccination Rates Among Parents and Health Care Personnel in a German Neonatology Department

**DOI:** 10.3390/vaccines6010003

**Published:** 2018-01-05

**Authors:** Horst Buxmann, Anne Daun, Sabine Wicker, Rolf Lambert Schlößer

**Affiliations:** 1 Department for Children and Adolescents, Division for Neonatology, University Hospital Frankfurt, Goethe University, Theodor-Stern-Kai 7, D-60590 Frankfurt/Main, Germany; anne.daun@kgu.de (A.D.); rolf.schloesser@kgu.de (R.L.S.); 2 Occupational Health Service, University Hospital Frankfurt, Goethe University, Theodor-Stern-Kai 7, D-60590 Frankfurt, Germany; sabine.wicker@kgu.de

**Keywords:** influenza vaccine, vaccination rates, pregnancy, neonate, preterm, neonatal intensive care unit, health care personnel

## Abstract

The influenza vaccination is recommended for all German pregnant women and health care personnel (HCP). We are the first to publish vaccination rates of mothers of hospitalized newborns and HCP in neonatal units. Between September 2016 and March 2017, data were collected in our level-III neonatology department in this descriptive multidisciplinary study, using an anonymous questionnaire. As a result, 513 persons were asked to participate, including 330 parents and 183 HCP. We received an 80.3% (412/513) response rate, 87.3% (288/330), and 67.8% (124/183) from parents and HCP, respectively. Ten percent (16/160) of mothers and 4.7% (6/127) of fathers had been vaccinated in 2016–2017 and 54.4% (87/160) mothers and 52.2% (66/127) fathers ever in their lifetime. In 2016–2017, 51.2% (21/41) of physicians had been vaccinated, 25.5% (14/55) of nurses, and 50.0% (14/28) of other staff members. When comparing those who had more than five influenza vaccinations in their life time, physicians were at 43.9% (18/41) versus nurses at 10.9% (6/55) (*p* < 0.01), and other HCP at 7.4% (2/27) (*p* < 0.01). The influenza vaccine uptake rate of 10% in mothers of hospitalized neonates is disappointingly low, resulting in 90% of hospitalized neonates being potentially vulnerable to influenza infection at a time where the risk for influenza-related complication can be severe.

## 1. Introduction

Current German guidelines [1] recommend the influenza vaccination for all pregnant women without contraindications. This recommendation includes all health care personnel (HCP) and people who could be an origin of infection to persons-at-risk for a severe course of influenza disease. Preterm infants, particularly very low birth weight infants, are likely to develop severe influenza infections, and related complications, due to their immature immune systems, including deficits in B-cell function [2]. Therefore, both parents of preterm infants should be vaccinated against influenza during the season. The vaccine uptake rate in this area was not known. To address this gap in knowledge, we evaluated the vaccination rates among parents and the HCP of patients in our level-III neonatology department.

## 2. Materials and Methods

### 2.1. Study Design and Participants

In this descriptive multidisciplinary study, an anonymous questionnaire about active vaccination against influenza was provided to parents of hospitalized neonates and HCP with contact to these patients, after written informed consent was obtained, at the level-III neonatology department of the University Hospital Frankfurt/Main, Germany between 1 September 2016 and 31 March 2017. To preserve anonymity, the recirculation of the innominate questionnaires occurred by using closed letter boxes in the wards. 

### 2.2. Questionnaire

The questionnaire for the parents and HCP included the following nine questions: (1) If the person has been vaccinated against influenza in the 2016/2017season? (2) If the person has ever been vaccinated against influenza and how often? (3) Why the person would let themselves be vaccinated against influenza? (4) If the person thinks that the vaccination against influenza is effective? (5) Reasons why a person would not let themselves be vaccinated. (6) Would the person let themselves be vaccinated against influenza if they had a better knowledge about this vaccination? (7) The person’s estimated likelihood of catching the flu in the next season if they are not vaccinated? (8) If the person thinks that influenza is an illness which can be severe? (9) About the persons attitude concerning vaccinations in general. Additionally, the parents were asked if they were the mother or the father of the neonate, and the HCP were asked about their gender and their profession within the health care system: physician, nurse, or other profession. 

### 2.3. Statistical Analysis

Data collection and the descriptive analysis were conducted with Microsoft Excel 2010 (Microsoft Corp., Redmond, Washington, DC, USA). Differences in dichotomous variables were calculated with Fisher’s exact test. *p*-values less than 0.05 were considered as statistically significant. Statistical analysis was performed with BIAS 11.6 by Hanns Ackermann, Biomathematics, Johann Wolfgang Goethe University, Frankfurt/Main, Germany. 

### 2.4. Ethics

This study was approved by the Ethics Committee of the University Hospital Frankfurt, Goethe University, Germany (reference number: 177/16).

## 3. Results

**Figure 1:**
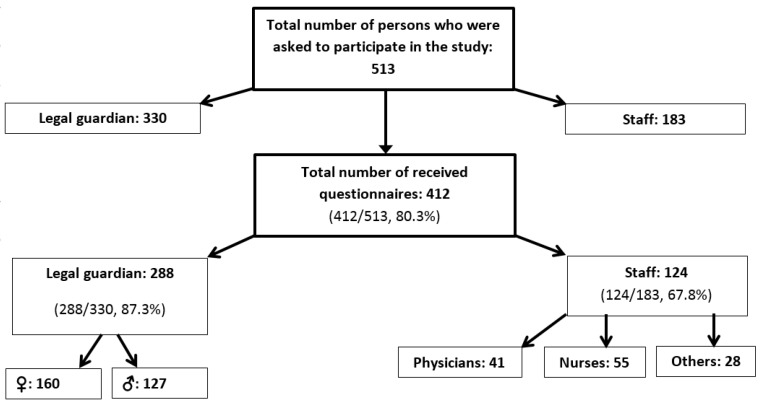
Flow chart of the recruitment process for the study.

During the study period, 513 persons were informed about, and asked to participate in the study, of which 330 were parents, representing 47.8% of 690 eligible parents and 183 were HCP representing 90.6% of 202 eligible HCP. We received back 80.3% (412/513) of the questionnaires: 87.3% (288/330) from all parents, 55.7% (160/287), of which were female, 44.3% (127/287) were male; and one survey with no gender information. We received 67.8% of the surveys (124/183) provided to all HCP, including 44.3% (55/124) from nurses, 33.1% (41/124) from physicians; and, 22.6% (28/124) from other staff-members like physiotherapists, paramedics, and cleaning personnel, for example (Figure 1).

Not all of the participants answered all the questions, with a maximum of three lacking answers per questionnaire. The missing answers are specified in Table 1. Ten percent (16/160) of the mothers and 4.7% (6/127) of the fathers whose neonates were patients in our department had been vaccinated in the 2016–2017 season. In terms of having been vaccinated ever, at least once, 54.4% (87/160) of the mothers and 52.2% (66/127) of the fathers responded with an affirmative to this question.

These vaccination rates are significantly lower than the 39.9% (49/124; *p* < 0.01) of vaccinated HCP in the 2016–2017 season and significantly lower than the lifetime HCP vaccination rate of 74.8% (92/123; *p* = 0.03). 

Focusing on the HCP, 90.2% (37/41) of physicians showed a tendency to be more often vaccinated in their lifetime, at least once, when compared to other staff-members with 77.8% (21/27), and nurses with 61.8% (34/55). Physicians were also more likely to be vaccinated in the 2016–2017 season, at 51.2% (21/41), when compared to other staff members at 50.0% (14/28), and nurses at 25.5% (14/55). These differences were statistically not significant. In terms of receiving more than five influenza vaccinations in their lifetime, physicians, at 43.9% (18/41), were vaccinated significantly more often than nurses, at 10.9% (6/55; *p* < 0.01), and other HCP at 7.4% (2/27; *p* < 0.01). 

The motivation for parents and HCP to allow for themselves to be vaccinated was predominantly to protect the neonates, followed by self-protection, and protection for other family members and friends (Table 1). A minority, more HCP than parents, believed in the effectiveness of active vaccination against influenza. Within the HCP group, significantly more physicians and other staff members believed that this vaccination is efficacious when compared to nurses (physicians vs. nurses; *p* = 0.02). The most often named reason why a person would not allow themselves to be vaccinated, was “no expected specific risk” for parents with 37.5% (95/253). Interestingly, four mothers stated that they did not allow themselves to be vaccinated against influenza, because they had been pregnant. For the HCP group, expected “missing efficacy” of the vaccination was the most named factor to not allow themselves to be vaccinated, in which nurses choose this argument significantly more often than physicians (69.0% (29/42) vs. 22.2% (4/18); *p* = 0.04).

If they had better knowledge about the influenza vaccination, more parents than HCP would allow themselves to be vaccinated. This difference was highly statistically significant when mothers were compared to nurses (*p* < 0.01). The estimated likelihood of catching the flu in the next season if not vaccinated was significantly higher in the HCP group when compared to the parents group (*p* < 0.01). Significantly more HCP agreed to the statement that “influenza is an illness that could take a severe course” compared to parents (*p* = 0.02). Within the HCP group, significantly more physicians agreed to this declaration when compared to nurses with (*p* < 0.01). Concerning vaccinations in general, HCP favor vaccinations compared to parents (*p* < 0.01). Within the HCP group, the support of vaccinations in general was significantly higher in physicians and other HCP, as compared to nurses (*p* < 0.05). The questions and answers are shown in Table 1.

## 4. Discussion

### 4.1. Parents

To our knowledge, this is the first study on influenza vaccination rates among parents whose neonates are hospitalized in a level-III neonatology department. The vaccination rate found for 2016–2017 of 10.0% in mothers was very low and at 4.7% in fathers, was even lower. Given the current recommendation of the German Standing Committee on Vaccination [1] to vaccinate all pregnant women against influenza, these data are disappointing. Unfortunately, a comparable 7% vaccination coverage in pregnant women was also found by Gaudelus et al. [3] in France, where the influenza vaccination is also recommended for all pregnant women. As a result, approximately 90% of the mothers from hospitalized term and preterm newborns in our cohort missed the chance to gain the well-described benefits of the influenza vaccination during pregnancy for their own health and that of their offspring [4]. The latter provides two benefits from the influenza vaccination of the mother: a reduced risk that the mother will be infected with influenza and transfers the virus to her baby, and the passive in utero immunization by materno-fetal influenza antibody transfer after vaccination during pregnancy [5,6]. 

Why are the vaccination rates among pregnant women so low? It seems to be a combination of a lack of information, an underestimated risk of influenza infection, fear of adverse events, and doubt in the efficacy of the influenza vaccination. Nearly 40% of all the legal guardians answered that they would allow themselves to be vaccinated if they would have had a better knowledge about this vaccination. Four women in our cohort did not let themselves be vaccinated because they were pregnant, which is an indication for this vaccination, recommended by the German Standing Committee on Vaccination (STIKO) [1]. The most-provided statement by parents in question number five was that they do not perceive a risk of being infected with influenza. With respect to the vaccination itself, only a quarter of the parents believed in the efficacy and nearly one-third were afraid of unexpected side effects of this vaccination. Although many studies have shown that the influenza vaccination during pregnancy is safe [4,7,8], the reported efficacy of about 50% in pregnant women [9] indicates considerable room for vaccine improvement. 

### 4.2. Health Care Personnel

The vaccination rates among HCP (39.5%) was significantly higher when compared to parents (7.6%), both in the 2016–2017 season (*p* < 0.01), and at least once in their lifetime (74.8% vs. 53.5%, respectively; *p* = 0.03). In comparison to other pediatric departments, these vaccination rates among the HCP are higher [10] or comparable [11]. Given the perceptions of parents of pediatric patients about influenza vaccinations in HCP, 76% of the parents felt that all HCP should be vaccinated [12], to benefit the HCP themselves and their patients [13], along with the strong evidence-based recommendation for the influenza vaccination of HCP [1,14], these results could be improved upon. 

The influenza vaccination rates within the HCP group were heterogeneous, showing the highest percentages for physicians both in the 2016–2017 season at 51.2% and 90.2% ever. The lowest rates were reported for nurses with 25.5% in the 2016–2017 season and 61.8% ever. This difference culminated in the highly significant difference concerning five or more influenza vaccinations ever, with 43.9% of all physicians and 10.9% of all nurses (*p* < 0.01), responding to this question in the affirmative. These findings align with those of Cozza et al. [10] and Pettke et al. [11] who also found higher vaccination rates among pediatric physicians when compared to pediatric nurses. This may be because significantly more physicians (46.3%) trust in the efficacy of the influenza vaccination than nurses (18.2%, *p* = 0.02). Additionally, significantly more physicians stated that “influenza is an illness which could take a severe course” at 52.5% compared to 13.5% of nurses (*p* < 0.01) and physicians tend to be less afraid of unexpected side effects as compared to nurses, at 33.3% vs. 47.6%, respectively (n.s.). Ofstead et al. [15] also found feared side effects (57.1%), when considering the risk of influenza infection as low (44.4%), and perceived poor vaccine effectiveness (31.3%) as the main reasons for HCP to not get vaccinated against influenza. 

The initiated vaccination promotion toolkit by Cozza et al. [10] unfortunately did not overcome their poor vaccination coverage of 14.2% in the five-year average in their pediatric department. Ofstead et al. [15] stated that “Strategies other than educational interventions are needed to increase influenza vaccination rates, and thereby to ensure healthcare worker and patient safety”. Unfortunately, there are no strategies published in the current literature with reliable boosted influenza vaccination rates in HCP of pediatric departments.

### 4.3. Limitations of the Study

Some limitations exist in the interpretation of the results of this study. Since the survey was based on a convenience sample by a self-administered questionnaire, the findings may not be generalizable to the general population of parents and HCP in other settings. Moreover, only 67.8% of staff members and 87.3% of the parents returned the questionnaire. Unvaccinated persons may have been less motivated to complete this survey [16], and vaccination rates may be overestimated because of this non-response bias. Furthermore, we do not know whether respondents had influenza disease before and how the experience of the flu could affect their willingness to be vaccinated. In addition, some participants did not answer all questions, which also limited the accuracy of the data.

## 5. Conclusions

To our knowledge, this is the first study on influenza vaccination rates among parents and health care personnel in a neonatology department. The 10% influenza vaccine rate of former pregnant women whose neonates were hospitalized is disappointingly low. About 90% of pregnant women miss the unique opportunity to protect two individuals with one vaccination, by actively protecting themselves and passively protecting their hospitalized neonates. The vaccination rates in HCP of 39.5% in the 2016–2017 season, and 74.8% in their lifetime, are comparable or even better than those found in other pediatric departments [10,11]. Physicians with 43.9% had significantly higher rates on more than five influenza vaccinations in their lifetime than nurses (10.9%) and other HCP (7.4%). There is much room for improvement. 

## Figures and Tables

**Table 1:** All questions and answers provided in response to the vaccination questionnaire.

Question No. 1: Have You been Vaccinated Against Influenza in the Actual Season (September 2016 to April 2017)?	Parents	Mothers	Fathers	Health Care Personnel	Physicians	Nurses	Other Health Care Personnel
Yes	22/288 (7.6%)	16/160 (10%)	6/127 (4.7%)	49/124 (39.5%)	21/41 (51.2%)	14/55 (25.5%)	14/28 (50.0%)
No	266/288 (92.4%)	144/160 (90%)	121/127 (95.3%)	75/124 (60.5%)	20/41 (48.8%)	41/55 (74.5%)	14/28 (50.0%)
No answer to this question	0/288	0/160	0/127	0/124	0/41	0/55	0/28
**Question No. 2: Have You Ever been Vaccinated Against Influenza?**							
Never	134/288 (46.5%)	73/160 (45.6%)	61/127 (48.0%)	31/123 (25.2%)	4/41 (9.8%)	21/55 (38.2%)	6/27 (22.2%)
Once	83/288 (28.8%)	44/160 (27.5%)	38/127 (29.9%)	26/123 (21.1%)	6/41 (14.6%)	12/55 (21.8%)	8/27 (29.6%)
2–5 times	55/288 (19.1%)	33/160 (20.6%)	22/127 (17.3%)	40/123 (32.5%)	13/41 (31.7%)	16/55 (29.1%)	11/27 (40.7%)
More than 5 times	16/288 (5.6%)	10/160 (6.3%)	6/127 (4.7%)	26/123 (21.1%)	18/41 (43.9%)	6/55 (10.9%)	2/27 (7.4%)
No answer to this question	0/288	0/160	0/127	1/124 (0.8%)	0/41	0/55	1/28 (3.6%)
**Question No. 3: Why Would You Let Yourself Vaccinate Against Influenza? (More Than One Answer Possible)**							
Self-protection	155/241 (64.3%)	87/132 (65.9%)	67/108 (62.0%)	82/124 (66.1%)	31/40 (77.5%)	29/38 (76.3%)	22/26 (84.6%)
Family and friends	160/241 (66.4%)	84/132 (63.6%)	76/108 (70.4%)	68/124 (54.8%)	29/40 (72.5%)	24/38 (63.2%)	15/26 (57.7%)
Children/patients	185/241 (76.8%)	102/132 (77.3%)	83/108 (76.9%)	92/124 (74.2%)	39/40 (97.5%)	32/38 (84.2%)	21/26 (80.8%)
No answer to this question	47/288 (16.3%)	28/160 (17.5%)	19/127 (15.0%)	20/124 (16.1%)	1/41 (2.4%)	17/55 (30.9%)	2/28 (7.1%)
**Question No. 4: Do You Think the Vaccination Against Influenza is Effective?**							
Yes	74/285 (26.0%)	42/159 (26.4%)	31/125 (24.8%)	45/124 (36.3%)	19/41 (46.3%)	10/55 (18.2%)	15/28 (53.6%)
No	19/285 (6.7%)	11/159 (6.9%)	8/125 (6.4%)	13/124 (10.5%)	0/41	12/55 (21.8%)	1/28 (3.6%)
Partly	126/285 (44.2%)	75/159 (47.2%)	51/125 (40.8%)	63/124 (50.8%)	21/41 (51.2%)	31/55 (56.4%)	11/28 (39.3%)
Don’t know	66/285 (23.2%)	31/159 (19.5%)	35/125 (28.0%)	4/124 (3.2%)	1/41 (2.4%)	2/55 (3.6%)	1/28 (3.6%)
No answer to this question	3/288 (1.0%)	1/160 (0.6%)	2/127 (1.6%)	0/124	0/41	0/55	0/28
**Question No. 5: Give Reasons, Why You Would Not Let Yourself Get Vaccinated Against Influenza?**							
Planned to get vaccinated, but not done yet	42/253 (16.6%)	23/136 (16.9%)	19/116 (16.4%)	14/73 (19.2%)	8/18 (44.4%)	5/42 (11.9%)	1/13 (7.6%)
No specific risk	95/253 (37.5%)	49/136 (36.0%)	45/116 (38.8%)	19/73 (26.0%)	2/18 (11.1%)	14/42 (33.3%)	3/13 (23.1%)
No severe illness	59/253 (23.3%)	34/136 (25.0%)	25/116 (21.6%)	8/73 (11.0%)	1/18 (5.6%)	5/42 (11.9%)	2/13 (15.4%)
No protective effect	60/253 (23.7%)	32/136 (23.5%)	28/116 (24.1%)	38/73 (52.1%)	4/18 (22.2%)	29/42 (69.0%)	5/13 (38.5%)
Afraid of unexpected side effects	78/253 (30.8%)	43/136 (31.6%)	35/116 (30.2%)	28/73 (38.4%)	6/18 (33.3%)	20/42 (47.6%)	3/13 (23.1%)
Afraid of injections	8/253 (3.2%)	4/136 (2.9%)	4/116 (3.4%)	1/73 (1.4%)	1/18 (5.6%)	0/42	0/13
Causes influenza	56/253 (22.1%)	30/136 (22.1%)	26/116 (22.4%)	19/73 (26.0%)	2/18 (11.1%)	14/42 (33.3%)	3/13 (23.1%)
No time	31/253 (12.3%)	10/136 (7.4%)	21/116 (18.1%)	8/73 (11.0%)	7/18 (38.9%)	1/42 (2.4%)	1/13 (7.7%)
Forgotten	26/253 (10.3%)	11/136 (8.1%)	15/116 (12.9%)	8/73 (11.0%)	2/18 (11.1%)	1/42 (2.4%)	4/13 (30.8%)
Doctor advised against it.	36/253 (14.2%)	19/136 (14.0%)	17/116 (14.7%)	4/73 (5.5%)	0/18	0/42	4/13 (30.8%)
No answer to this question.	35/288 (12.2%)	24/160 (15.0%)	11/127 (8.7%)	51/124 (41.1%)	23/41 (56.1%)	13/55 (23.6%)	15/28 (53.6%)
**Question No. 6: Would You Let Yourself Get Vaccinated Against Influenza if You Had a Better Knowledge About This Vaccination?**							
Yes	104/264 (39.4%)	61/146 (41.8%)	43/117 (36.8%)	20/83 (24.1%)	9/22 (40.9%)	2/41 (4.9%)	9/20 (45.0%)
No	47/264 (17.8%)	31/146 (21.2%)	16/117 (13.7%)	34/83 (41.0%)	9/22 (40.9%)	21/41 (51.2%)	5/20 (25.0%)
Don’t know	112/264 (42.4%)	53/146 (36.3%)	58/117 (49.6%)	28/83 (33.7%)	4/22 (18.2%)	17/41 (41.5%)	6%20 (30.0%)
No answer to this question.	24/288 (8.3%)	14/160 (8.8%)	10/127 (7.9%)	41/124 (33.1%)	19/41 (46.3%)	14/55 (24.5%)	8/28 (28.6%)
**Question No. 7: How Big is the Estimated Likelihood to Catch the Flu in the Next Season If You are Not Vaccinated?**							
No risk	32/280 (11.4%)	12/157 (7.6%)	19/122 (15.6%)	7/117 (6.0%)	0/38	6/52 (11.5%)	1/27 (3.7%)
Almost no risk	52/280 (18.6%)	30/157 (19.1%)	22/122 (18.0%)	14/117 (12.0%)	1/38 (2.6%)	8/52 (15.4%)	5/27 (18.5%)
Little risk	87/280 (31.1%)	48/157 (30.6%)	39/122 (32.0%)	23/117 (19.7%)	9/38 (23.7%)	10/52 (19.2%)	4/27 (14.8%)
moderate	79/280 (28.2%)	47/157 (29.9%)	32/122 (26.2%)	52/117 (52.4%)	17/38 (44.7%)	24/52 (46.2%)	11/27 (40.7%)
Quite big	18/280 (6.4%)	13/157 (14.6%)	5/122 (4.1%)	13/117 (11.1%)	5/38 (13.2%)	3/52 (5.8%)	5/27 (18.5%)
Big	8/280 (2.9%)	4/157 (2.5%)	4/122 (3.3%)	3/117 (2.6%)	2/38 (5.3%)	1/52 (1.9%)	0/27
Very big	4/280 (1.4%)	3/157 (1.9%)	1/122 (0.8%)	5/117 (4.3%)	4/38 (10.5%)	0/52	1/27 (3.7%)
No answer to this question.	8/288 (2.8%)	3/160 (1.9%)	5/127 (3.9%)	7/124 (5.6%)	3/41 (7.3%)	3/55 (5.5%)	1/28 (3.6%)
**Question No. 8: Influenza is an Illness Which could Take a Severe Course.**							
Strongly disagree	5/279 (1.8%)	3/157 (1.9%)	2/122 (1.6%)	0/121	0/40	0/52	0/28
Disagree	9/279 (3.2%)	5/157 (3.2%)	4/122 (3.3%)	1/121 (0.8%)	0/40	1/52 (1.9%)	0/28
Disagree in most parts	9/279 (3.2%)	5/157 (3.2%)	4/122 (3.3%)	3/121 (2.5%)	1/40 (2.5%)	1/52 (1.9%)	2/28 (7.1%)
Neutral	80/279 (28.7%)	43/157 (27.4%)	37/122 (30.3%)	13/121 (10.7%)	2/40 (5.0%)	6/52 (11.5%)	4/28 (14.3%)
Agree in most parts	60/279 (21.5%)	31/157 (19.7%)	29/122 (23.8%)	16/121 (13.2%)	3/40 (7.5%)	10/52 (19.2%)	3/28 (10.7%)
Agree	79/279 (28.3%)	49/157 (31.2%)	30/122 (24.6%)	54/121 (44.6%)	13/40 (32.5%)	27/52 (51.9%)	13/28 (46.4%)
Strongly agree	37/279 (13.3%)	21/157 (13.4%)	16/122 (13.1%)	34/121 (28.1%)	21/40 (52.5%)	7/52 (13.5%)	6/28 (21.4%)
No answer to this question.	9/288 (3.1%)	3/160 (1.9%)	5/127 (3.9%)	3/124 (2.4%)	1/41 (2.4%)	2/55 (2.6%)	0/28
**Question No. 9: Which Attitude Concerning Vaccinations in General Do You Agree Most with?**							
Totally against	2/278 (0.7%)	1/156 (0.6%)	1/127 (0.8%)	0/122	0/40	0/54	0/28
Mostly against	3/278 (1.1%)	2/156 (1.3%)	1/127 (0.8%)	2/122 (1.6%)	0/40	2/54 (3.7%)	0/28
Partly against	23/278 (8.3%)	16/156 (10.3%)	7/127 (5.5%)	6/122 (4.9%)	0/40	5/54 (9.3%)	1/28 (3.6%)
Neutral	81/278 (29.1%)	35/156 (22.4%)	46/127 (36.2%)	9/122 (7.4%)	0/40	7/54 (13.0%)	2/28 (7.1%)
Partly for	73/278 (26.3%)	46/156 (29.5%)	27/127 (21.3%)	22/122 (18.0%)	2/40 (5.0%)	14/54 (25.9%)	6/28 (21.4%)
Mostly for	74/278 (26.6%)	40/156 (25.6%)	34/127 (26.8%)	40/122 (32.8%)	15/40 (37.5%)	18/54 (33.3%)	7/28 (25.0%)
Totally for	22/278 (7.9%)	16/156 (10.3%)	6/127 (4.7%)	43/122 (35.2%)	23/40 (57.5%)	8/54 (14.8%)	12/28 (42.9%)
No answer to this question	10/288 (3.5%)	4/160 (2.5%)	5/127 (3.9%)	2/124 (1.6%)	1/41 (2.4%)	1/55 (1.8%)	0/28
